# Does Oral Endotoxin Contribute to Systemic Inflammation?

**DOI:** 10.3389/froh.2022.911420

**Published:** 2022-05-23

**Authors:** Camille Zenobia, Richard P. Darveau

**Affiliations:** ^1^Os Salutem LLC, Hampton, NJ, United States; ^2^Departments of Periodontology and Microbiology, University of Washington, Seattle, WA, United States

**Keywords:** metabolic endotoxemia, *Porphyromonas gingivalis*, periodontal disease, gut dysbiosis, systemic inflammation

## Abstract

The oral microbiome, with a unique emphasis on *Porphyromonas gingivalis* has been associated with a constellation of inflammatory diseases such as cardiovascular disease, rheumatoid arthritis, Alzheimer's disease, type II diabetes, and non-alcoholic associated fatty liver disease. Periodontal disease has also been shown to induce “leaky gut” leading to metabolic endotoxemia. Several recent studies investigating the habitants of the blood microbiome have found the majority of species appear to be derived from oral and skin bacterial communities in otherwise healthy individuals. Many of the same pathologies associated with perturbations of oral health, such as cardiovascular disease, show alterations to the composition of the blood microbiome as well as circulating neutrophil phenotypes. Gingival inflammation is associated with activated blood neutrophil phenotypes that can exacerbate a distal inflammatory insult which may explain the connection between oral and systemic inflammatory conditions. While in the oral cavity, neutrophils encounter oral microbes that are adept in manipulating neutrophil activity which can re-enter the vasculature thereafter. Endotoxin from oral microbes can differ significantly depending on bacterial community and state of oral health to alter cellular LPS tolerance mechanisms which may contribute to the primed neutrophil phenotype seen in periodontitis and provide a mechanism by which the oral-microbes can affect systemic health outcomes. This review synthesizes the studies between inflammatory diseases and oral health with emphasis on microbiome and corresponding lipopolysaccharides in immune tolerance and activation.

## Introduction

Despite that oral microbiota or “animalcules” were the first bacteria of the human microbiome to be described with drawings by Antoine Leeuwenhoek (inventor of the microscope) much of what we know about the human microbiome has been dictated by study of the microbial community as it pertains to gastrointestinal health. Our understanding of the human microbiome has evolved from thinking of bacteria as invaders or freeloaders to now considering them a necessary and active participant in human metabolic function. Recent investigation suggests a primary role for oral bacteria in driving gut-microbial dysbiosis leading to Inflammatory Bowel Disease, and metabolic endotoxemia [1]. Coinciding with these findings, research into characterization of the blood microbiome has shown the presence of the oral microbiome in the humoral system in otherwise healthy individuals. In dental-focused research, it has long been acknowledged that there is a link between oral health and systemic parameters of health but a direct, stepwise mechanism of action has not been elucidated that satisfies all inflammatory pathologies implicated. This review aims to synthesize the disparate study between blood microbiome, metabolic endotoxemia, and oral pathology to develop a new hypothesis and potential mechanism for the connection between oral and systemic inflammatory pathology.

## Porphyromonas Gingivalis and Systemic Connections

The gingival tissues in the oral cavity that surround each tooth are highly microbially colonized and have evolved with our host tissues over thousands of years. Correlations have been found between oral health and cardiovascular disease [2, 3], Stroke [4], pregnancy outcomes [5], Rheumatoid Arthritis [6], Alzheimer's disease [7], type II diabetes [8], Metabolic Syndrome [9], and Non-Alcohol Associated Fatty Liver Disease [10]. Many studies have looked to specific microbial inhabitants of the oral cavity to explain the link between localized oral pathology and systemic impacts. One of the primary suspects, *Porphyromonas gingivalis* (*P. gingivalis*) has been an ideal candidate of pathological etiology with ability to induce and feed off of inflammatory mediators [11], survive intracellular conditions and travel via host cells to remote tissues [12], alter lipopolysaccharide (LPS) structures to evade host recognition [13]. More importantly, *P. gingivalis* has been linked to the etiology of nearly every systemic pathology that has been described as an inflammatory, systemic comorbidity of periodontitis [14, 15].

*P. gingivalis* has been shown to disseminate systemically either from direct release into blood through periodontal lesions or by cell to cell invasion [16–18]. Recent characterization of a human blood microbiome has shown a small but viable population of bacteria originating primarily from both oral and skin microbial communities, including *P. gingivalis* in otherwise healthy individuals [17, 18]. The bacteria of the blood microbiome are primarily found in the Buffy coat, implicating white blood cells as the main vehicle of dissemination. Concurrent investigation into metabolic endotoxemia has illuminated a role for oral bacteria, specific to periodontal pathology, as a driver for the induction of microbial dysbiosis of the gut and subsequent dissemination of enteric lipopolysaccharide (LPS) into the blood reviewed here [19]. The resulting scenario with blood containing oral microbes that include *P. gingivalis*, and enteric-derived LPS could result in very different inflammatory responses depending on the state of oral health since gingival inflammation has been shown to prime circulating neutrophils [20].

## Oral Microbiome: Acquisition to Dysbiosis

To set the stage for gingival inflammation, it is necessary to review the basics of oral biofilm succession in order to more fully discuss the nuances of the host-microbe relationship. The oral cavity is diverse in hard and soft tissue types as well as microbial biofilm diversity reflecting over 400 unique bacterial taxa [21]. The epithelium of the soft tissues, namely the junctional epithelium, that form around hard tissues (teeth) do not contain tight junctions and are proximal to the highly vascularized gingiva which allows a constant supply of innate immune cells to the gingival crevice to patrol, and control microbial load [21, 22]. The sulcus, or gingival pocket contains a very well described microbial composition that develops in a stepwise manner starting with the deposition of salivary proteins on the tooth surface, bacterial adhesins, and bacteria nutrient demands shape the development of the oral biofilm [23]. The organization of the oral biofilm is dependent on bacterial relationships that have been described and categorized into disparate complexes that, once formed, allow the biofilm to grow and form increasingly complex structures [24]. The bacterial members that comprise the early colonizers of the gingival crevice are those with the capacity to adhere to the pellicle of the tooth and are considered only moderately pathogenic and primarily Gram-positive. The resulting foundation of early biofilm allows access to a bridging community of bacteria known as the orange complex to form which have been found to be increasingly capable of causing periodontal pathology (e.g., *F. nucleatum*). Finally, the “Red complex” of bacteria can gain entry, these members are primarily Gram-negative, contain endotoxin, and are well described for their highly pathogenic features (e.g., *P. gingivalis*). The presence of “Red complex” bacteria, or keystone pathogens can cause an outgrowth of commensal microbiota that results in increased microbial burden, inflammatory pathology, and tissue destruction [25]. This classical model of microbial succession is under constant revision as we evolve our understanding of the coordinated nutritional-interplay and adherence mechanisms between microbial species.

While the periodontal-associated pathogens can remain low in abundance compared to the overall microbiome community, they can still expand in number and alter their phenotype to become increasingly pathogenic. As the microbial burden increases, so too does the inflammatory environment. The cytokine milieu and organization of the microbial community can influence the activity and expression of pathogenic features of the oral pathobionts. One example is the lipid A moiety from *P. gingivalis* LPS which can be altered when exposed to increase in hemin, temperature, or co-culture with *F. nucleatum* resulting in unique LPS phenotypes with varying capacity to inhibit Toll-Like-Receptor-4 (TLR4) activity [3, 26, 27]. *P. gingivalis* is considered a keystone pathogen and an etiological agent in the development of periodontitis [25]. During the progression of localized periodontal inflammation, the local gingival environment changes to potentiate a phenotype alteration in the lipid-A portion of the LPS structure. Indeed, these changes have been shown to occur in a natural periodontitis human infection; patients with periodontal disease clinically evaluated for LPS activity from plaque harvested from sites of either active disease or healthy pockets. The LPS activity was shown to be TLR4-antagonist for sites with active disease whereas otherwise healthy pockets were found to be TLR4-agonist [3, 28]. The alteration to LPS signaling in gingival pockets of periodontitis has the potential to differentially prime responding neutrophils and disrupt the LPS-tolerance mechanism [29]. While *P. gingivalis* has been shown to disseminate beyond the oral cavity within leukocytes or directly into vasculature, the potential for this bacteria to induce local neutrophil-specific LPS priming in the gingival crevice might be another way that the pathobiont can influence distal inflammation.

Oral endotoxin has been a feature of extraordinary inflammation in gingival tissues, capable of causing exacerbated inflammation in leukocyte adhesion deficiency (LAD-1) patients that lends to a distinct microbial community [30]. The neutrophils in these LAD-1 patients are unable to migrate through gingival tissues to control the microbial burden, but the localized inflammatory response is sufficient to prevent microbial infiltration into the gingiva despite the increasing microbial load and translocation of LPS into gingival tissue. The microbial community that develops in severe LAD-1 is unique from the bacterial community associated with Localized Aggressive Periodontitis (LAP) indicating a specialized relationship exists between the inflammatory etiology and resulting microbiome [30]. A similar observation has been shown in the oral microbiome from receptor knockout mice. Mice deficient in either TLR-2,−4 responsible for host-recognition of bacterial cell wall lipoproteins and LPS, were found to develop different microbial communities and decreased neutrophil recruitment [31]. Furthermore, the periodontitis that develops from *P. gingivalis* is TLR4 dependent unlike disease induced by ligature [31, 32]. Together, these data suggest that host-signaling and microbial communities are in lock-step as they respond to one another. Further, the etiology of inflammatory insult can induce and be shaped by specific bacterial endotoxin to impart different health outcomes.

## Oral Neutrophil: Activation or Tolerance?

Preserving the integrity of the gingiva is paramount for the control of microbial load in the oral cavity. Neutrophils are the primary surveillance immune cell in the gingival tissues, innervating the permeable junctional epithelium with 30,000 neutrophils passing though per minute in healthy tissues [33]. Neutrophil migration into gingival tissues still occurs in the absence of the microbiota as seen in germ-free mice, perhaps lending a resilience to the epithelium where the neutrophil has been found to rescue epithelial cells from *P. gingivalis* induced-cell death [34, 35]. In healthy gingival tissues, the junctional epithelium remains in a low-differentiation state with a turnover rate of about 4–6 days [36]. As the oral biofilm builds and increases complexity in the gingival crevice, the mid- and late-colonizers are adept at manipulating neutrophil antimicrobial functions [37, 38]. The dysregulation of the neutrophil can exacerbate tissue damage and lead to chronic periodontal lesions, likely due to ineffective bacterial killing [39, 40]. During periodontitis, physical disruption to the epithelium, and microbiome alterations can impact tissue-neutrophil responses to cause a local-hyperinflammatory phenotype [41]. Evaluation of circulating neutrophils confirmed that people with periodontitis have the same hyperinflammatory phenotype in the blood which does not resolve with periodontal treatment [42]. This lack of resolution is perhaps due to the development of trained immunity where LPS exposure has been found to influence memory-like responses in bone marrow neutrophils [43]. Periodontal pathology causes alterations to the connective tissue and results in the apical migration of the junctional epithelium creating an exaggerated periodontal pocket and disorganized neutrophil recruitment [44]. The increase in neutrophils recruited to the oral cavity during gingivitis and periodontitis correlates predictably with pathology [45].

In oral tissue homeostasis, the relationship between gingival epithelial cells and neutrophil recruitment is tightly regulated with a CXCR2 signaling requirement for a healthy host-response to the commensal community that develops in the interdental region of the gingival tissues [34, 46]. The CXCR2 requirement is likely due to coordinated signaling between the neutrophil and endothelial cells which has been shown to be necessary for functional host response to LPS in the lung [47]. The tissue-neutrophil appears to respond differently to different LPS structures than neutrophils in circulation. To illustrate this phenomenon, the neutrophil response to LPS was evaluated in the presence or absence of platelets or serum [48]. Seven different bacterial LPS preparations were found to induce NETosis broadly when cells were cultured in the presence of platelets, whereas neutrophils grown in platelet- and serum-free conditions produced NETs to only two of seven the LPS structures [48]. Together, a picture emerges of a protective relationship between the epithelium and neutrophil that dampens the host response to LPS type when barrier functions are intact. The progression from health to periodontal disease results in the degradation of the junctional epithelium structure around the tooth and dysregulation of neutrophil response which likely results in the platelet-rich conditions that could allow the neutrophil to respond more aggressively to unique LPS structures. Indeed, it has been shown that a subpopulation of neutrophils exist in chronic periodontitis that exhibit a pro-inflammatory phenotype with increased NETosis as measured by myeloperoxidase and histone citrullination [41]. In summary, when barrier functions are compromised, the oral neutrophil response to LPS is likely altered lending to a hyperinflammatory phenotype which propagates to the circulation.

## LPS Stimulation: Prime, Tolerize, or Train

Continued exposure to LPS typically results in tolerance and is well described for repeated LPS exposure in innate immune cells, causing a downregulation of inflammatory cytokines [49]. Much of the research in LPS tolerance is uniquely *Escherichia coli* (*E. coli*) specific but the cellular response looks very different for *P. gingivalis* LPS where repeated exposure in neutrophils has been shown to increase IL-8, and phosphorylated JNK production [50]. Only one *P. gingivalis* LPS preparation was tested in this study whereas another study found other *P. gingivalis* preparations impact IL-8 differentially [51]. These findings raise questions around how neutrophil tolerance might be impacted by LPS alterations, different growth conditions, or periodontal pathology. Neutrophils isolated from patients with chronic periodontitis show an exaggerated cytokine response, including IL-8 in samples isolated from both saliva and blood [42, 52]. Bone-marrow derived neutrophils have been found to develop memory-like responses to repeat LPS exposure. Neutrophils primed with either low or high dose LPS and then re-challenged after a rest-period were either sensitized or tolerized, respectively, which also affected their migration and phagocytosis functions [43]. In macrophages, priming cells with either *E. coli* or *P. gingivalis* LPS differentially impacts tolerance during subsequent cross-LPS challenge [53]. Peritoneal derived macrophages primed with *P. gingivalis* LPS and then challenged with *E. coli* LPS produce significantly more TNFα and IL-10 than those challenged first with *E. coli* and then with *P. gingivalis*. It was found that *P. gingivalis* LPS priming led to upregulation of TLR2 genes whereas *E. coli* LPS priming upregulated TLR4 genes. These mechanisms of tolerance in mouse models appear to change during the aging process where aged mice show impared tolerance to both *E. coli* and *P. gingivalis* LPS [53]. Interestingly, aged mice also naturally develop GI dysbiosis [54], periodontitis [54, 55], and altered responses to induced metabolic endotoxemia [56] which complicates the understanding of how tolerance can be disrupted with age. It is also worth noting that *P. gingivalis* LPS is not the only contributor to oral endotoxin (LPS_o_) as other oral pathobionts and their respective LPS have been found to contribute to differential host responses alone or in combination [57] and will require additional consideration when comparing microbial profiles present in health or disease. Alterations to LPS structure can clearly impact tolerance functions which are likely to affect downstream events that occur during progression of periodontitis.

## LPS in Circulation

The disruption of barrier function and host response in the oral cavity can allow dissemination of oral bacteria and LPS into the vasculature that coincides with alterations to systemic neutrophil phenotypes. A study of induced gingivitis in humans found the induction of gingivitis corresponded with a hyperinflammatory blood neutrophil phenotype [20, 58]. In mice with induced periodontitis, the development of a hyperinflammatory blood-neutrophil was also described and found capable of inducing exacerbated pathology when mice were subsequently challenged with peritonitis [20, 58]. The authors very nicely show that oral pathology can impact innate inflammatory responses distal to gingival insult. Additional data revealed circulatory macrophages were unaffected by induction of periodontitis. It is notable that a ligature was utilized in this mouse model of periodontitis whereas other models of induction can produce different inflammatory responses [59]. It is not yet understood if the change to circulatory neutrophil phenotype is a result of direct priming from a local gingival lesion where neutrophils are the primary responder or by indirect exposure from the release of oral bacteria, or their PAMPS (e.g., LPS) to circulation. It has been found that simple tooth-brushing can cause septic conditions [60], allowing the release of oral bacteria into circulation but sans a periodontal insult, this exposure does not appear to be a factor in driving an inflammatory neutrophil phenotype since the resolution of gingivitis, by reinstatement of oral hygiene resulted in decrease to inflammatory phenotype [20, 58]. Thus, there exists a number of ways to prime the circulating neutrophil but there seems to be a unique relationship between an inflammatory periodontal insult and the resulting circulatory neutrophil phenotype that could set the stage for chronic inflammation.

**Figure 1:**
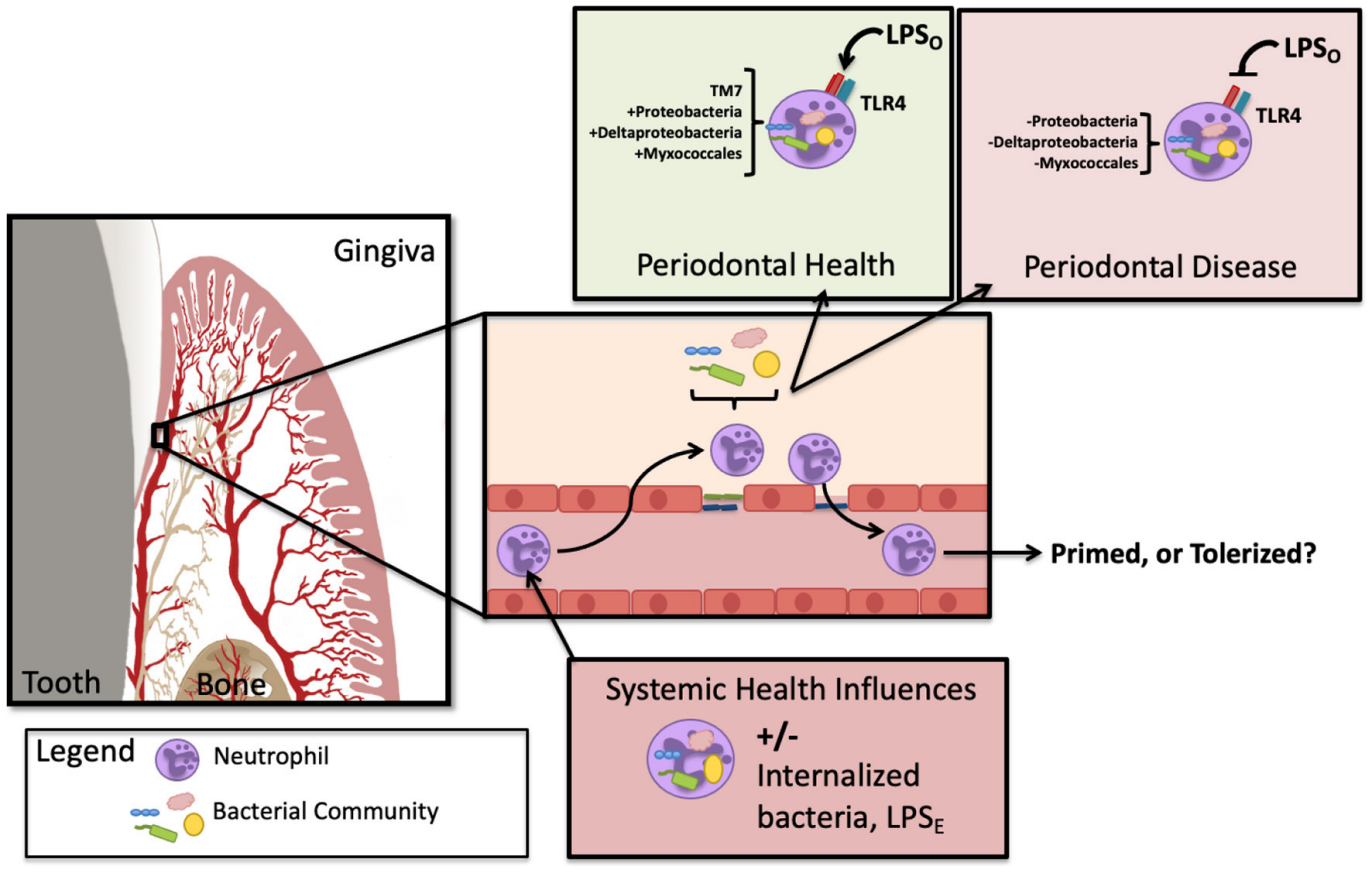
Oral endotoxin has the potential to prime or tolerize the infiltrating neutrophil. In circulation, the neutrophil may contain bacteria and/or be exposed to enteric LPS (LPS_E_) and subsequently migrate into tissues of the gingiva. Depending on the state of oral health, oral LPS (LPS_O_) can shift phenotypes with the changes that arise during microbial dysbiosis. Depending on the neutrophil state of activation, the subsequent agonism or antagonism of TLR4 could either prime or tolerize the migrating neutrophil. In addition, the circulating neutrophil has been found to harbor altered bacteria in periodontitis, illustrated here as described by Emery et al. [18].

**Figure 2:**
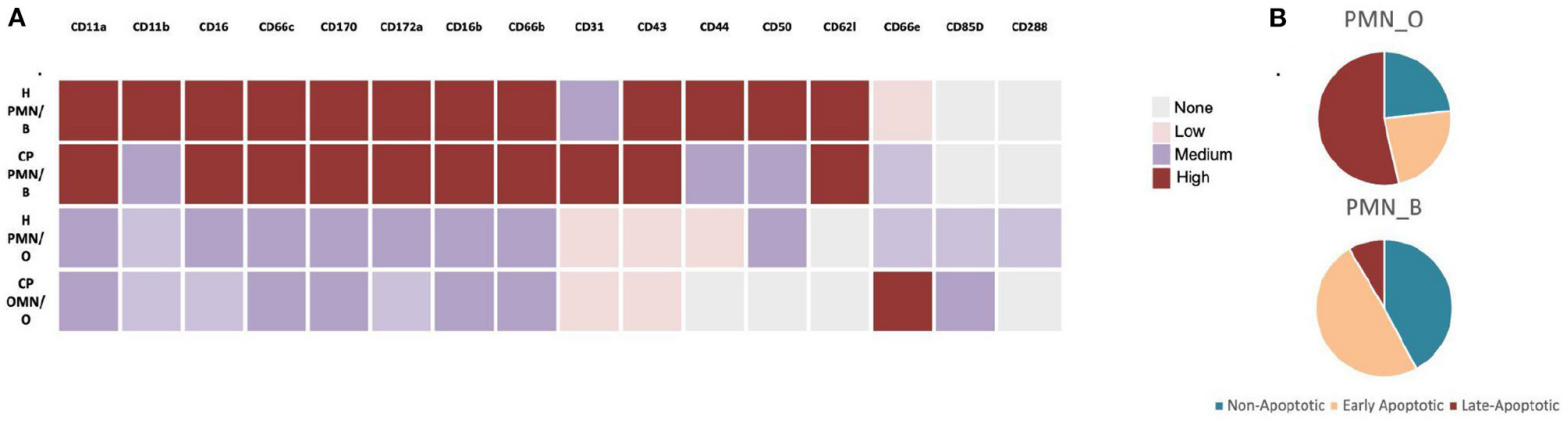
Oral vs. Blood Neutrophil Phenotypes. **(A)** Comparison of cluster differentiation expression levels of oral (O) and blood (B) neutrophils in oral- health (H) vs. chronic periodontal disease (CP). **(B)** Apoptotic profile of oral vs. blood neutrophils in otherwise healthy individuals. Illustrated here as described by [62, 63], respectively.

Consideration of the environmental conditions of the circulatory neutrophil that patrols the gingival tissue is key to unraveling the mystery of downstream inflammatory responses. If, for example, a high fat diet results in the outgrowth of oral bacteria which then creates gut-microbiome dysbiosis and leaky gut (as seen in models of metabolic endotoxemia discussed below), enteric endotoxin is now present in the circulation. In this scenario, the circulatory neutrophil is potentially primed with an *E. coli*-like LPS which could result in upregulation of TLR4 or even NETosis. If the same neutrophil is found migrating through the gingival tissue, the pathological potential for exaggerated inflammatory response could be very different if periodontal inflammation is present Figure 1. It is curious that oral hygiene can correct the circulating hyper-inflammatory neutrophil as seen during resolution of human-induced gingivitis [20, 58] whereas periodontal disease seems to crystallize the circulating neutrophil phenotype into a permanent feature [42], perhaps a function of a cross-tolerance issue from a changing LPS-environment in the gingival pocket. Figure 2 highlights the changes to neutrophil phenotypes in health and chronic periodontal disease, contrasting the neutrophil found in the oral cavity with those in circulation. It is understood that neutrophil exposure to LPS can result in tolerance or trained immunity, typically dictated by the dose which has been reviewed recently with respect to periodontal health, disease, and endotoxemia [61]. Less understood is to what extent the oral-derived LPS can participate in directing neutrophil immunity since the variable microbial load, resulting LPS dose, and LPS-phenotypes are relatively unexplored in the context of systemic health. LPS-induced trained immunity has shown an enhanced cellular response to secondary insult, similar to what the Glogauer group found in their 2-hit-animal study where mice with periodontal disease subsequently challenged with peritonitis were saddled with an exaggerated neutrophil response to the peritoneal cavity [20]. The LPS priming and tolerance mechanisms inherent to the circulating neutrophil may play a significant role in shaping trained immunity and also help explain why gingivitis is clinically reversible while periodontitis is not.

## Oral Health TO Metabolic Endotoxemia

Metabolic endotoxemia is defined by chronic subclinical levels of systemic LPS. The presence of LPS release into the circulatory system is thought to stem from an inflammatory pathology resulting from “leaky gut” that has been linked to type II diabetes, non-alcoholic associated fatty liver disease, and increased cardiovascular disease; recently reviewed here [64]. Enteric LPS is considered the pathological agent that causes metabolic endotoxemia, and thus, models seeking to recapitulate what is seen in clinical pathology utilize *E. coli* LPS for study [65–67]. Early observations found metabolic endotoxemia can develop from a high fat, low fiber diet. As clinical and animal models were established, the mechanism of pathogenesis implicated GI microbial dysbiosis along with disruption to intestinal epithelial cells, and mucous-layer to allow enteric LPS (LPS_E_) to enter the circulatory system unmitigated [64, 68]. Induction of metabolic endotoxemia in clinical and animal models has been primarily through a high fat, low fiber diet, or direct injection of *E. coli* LPS [65–67, 69] but subsequent study of metabolic endotoxemia and periodontitis has shown that ingestion of perio-pathogens can induce similar pathology [70, 71].

*P. gingivalis* has been implicated in pathogenesis of metabolic endotoxemia either by release of whole, inactivated bacteria directly into blood or indirectly by ingestion and subsequent induction of intestinal microbial dysbiosis [72–74]. In an attempt to understand the capacity of different bacteria to cause metabolic endotoxemia, a recent investigation of different bacterial LPS structures found induction was limited to *E. coli* LPS. The other LPS structures tested were either under-acylated or mono-phosphorylated and incapable of inducing the corresponding pathology; *P. gingivalis* LPS was included in the study and failed to induce blood glucose levels, a requirement for induction of metabolic endotoxemia [75]. Other studies have shown that *P. gingivalis* LPS can in fact induce disease when combined with a high fat diet [74]. These mice also developed Non-Alcoholic Fatty Liver Disease, thus further corroborating the link between the oral pathobiont, periodontitis and fatty liver. These data implicate an etiological role for periodontitis when coupled with a high fat diet for the development of metabolic endotoxemia pathology. Intriguingly, it has also been shown that feeding a mouse a high fat diet results in natural periodontitis [71]. It is not clear if the resulting GI microbial dysbiosis that occurs with high fat diet is due to the increased ingestion of perio-pathogens or the diet itself but the developing periodontal bone loss appears to be LPS dependant [76]. A more recent study showed that removal of one of two signaling components essential for LPS recognition, TLR4 and CD14, by utilization of knockout mice were both protected from diet-induced metabolic endotoxemia despite having the same weight gain as wild type, implicating a role for the LPS recognition, not diet, as an upstream requirement for induction of GI bacterial dysbiosis and corresponding “leaky-gut” [77]. The *P. gingivalis* gavage model of periodontal disease is distinct from the ligature-induced model [59] with the capacity to induce GI dysbiosis, and metabolic endotoxemia [72]. Careful comparison of these two models might shed light on how periodontal priming might affect systemic inflammatory responses. It does seem plausible that the combination of high fat diet induced periodontitis (seen in the mouse model) and subsequent introduction of an under-acylated or mono-phosphorylated LPS structure, like those of *P. gingivalis* to the circulation could induce metabolic endotoxemia pathology and increase the risk of developing other systemic inflammatory diseases. More work is needed to identify the origins and types of circulating LPS seen in clinical metabolic endotoxemia disease.

Systemic LPS is an obvious problem with regard to circulating neutrophils. Evaluation of blood neutrophils in experimental human endotoxemia, experimental gingivitis, periodontitis, cardiovascular disease, and type II diabetes all show significant alterations to neutrophil phenotype, activation, and function [52, 58, 78–80]. Fine et al. [20] nicely demonstrated that induction of periodontitis can selectively induce blood neutrophils to cause exacerbated disease in mice subsequently challenged with peritonitis. The neutrophil appears to be the common actor on the chronic inflammatory stage. It has been shown that when the circulatory neutrophil is endotoxin-tolerized, by administration of *E. coli* LPS, the tolerized neutrophil is more efficient at responding to secondary infection than a non-tolerized neutrophil [81]. TLR4, the main host-cellular-receptor for endotoxin recognition, is implicated in the pathogenesis in Rheumatoid Arthritis [82], cardiovascular disease [83], and type II diabetes [84]. TLR4 inhibition is currently being explored for treatment of Rheumatoid Arthritis [85] with varying success [86]. C. Genco's lab has explored the relationship between different LPS structures of *P. gingivalis* in a mouse model of atherosclerosis to find that the antagonist-structure allowed for bacterial survival and disease progression while the agonist-structure did not suggesting that the TLR4-response is protective [87, 88]. In type II diabetes, the TLR4 function appears dysfunctional with neutrophils exhibiting tolerance behavior when exposed to LPS [89]. Although the neutrophil appears central to each inflammatory pathology, so too does the specific TLR4-host-response ranging from protective to pathological. Deepening our understanding around the specific neutrophil-priming events by bacterial composition, including LPS-phenotypes by tissue location will greatly improve strategies for treatment and prevention.

## Neutrophils and the Blood Microbiome

The blood microbiome is yet another indicator linking inflammatory pathologies where a dysbiotic microbial profile has been found in Rheumatoid Arthritis [90, 91], Diabetes [92–94], cardiovascular disease [95], non-alcohol associated fatty liver disease [93], and periodontal disease [18] when compared to healthy controls. The circulatory neutrophils have been implicated as a “Trojan Horse” [96] with capacity to allow dissemination of bacteria that remain viable and pathogenic [97]. More recently, neutrophil-specific microbiomes have been identified within the categorical study of the blood microbiome [98, 99]. Despite early evidence to the contrary, the circulatory system has long been considered a closed, sterile compartment. Recent technological advances for microbial evaluation, primarily Next Generation Sequencing have allowed scientists to re-evaluate the blood with greater sensitivity and rigor. Although methodology is still tricky and nuanced, there is clear evidence that the blood is not a sterile environment and is subject to, at minimum, a transient population of bacteria or, more likely, an ever-present bacterial community capable of impacting systemic health. It is intriguing that many of the inflammatory pathologies have in common, periodontitis, changes to neutrophil phenotypes and skewed blood microbial profiles. The present foray into characterization of the blood microbiome may shed some light on how the steady presence of circulating bacteria, and neutrophil phenotypes may contribute to a secondary insult or chronic inflammation.

Prior to broad investigation into the blood microbiome, it has long been known that viable oral pathobionts can exist within the circulatory system [100]. Studies of arterial plaques from patients with cardiovascular disease have repeatedly identified bacterial lipids as well as whole bacteria derived from the oral microbiome, including *P. gingivalis* [12, 101]. It is notable that gut bacteria have also been found in arterial plaques [102]. A recent study of the blood microbiome from periodontally healthy and diseased found surprisingly little difference in total bacterial DNA abundance between healthy and disease profiles but did note the presence of *P. gingivalis* in both study groups [18]. It would be expected that those with periodontal inflammation would also have increased bacteremia, or bacterial load but the methodology used in this study selectively evaluated whole bacteria from white blood cells. Therefore, transient bacteria that were not housed in a host cell were not considered. This is unfortunate because the cell-constrained bacterial cells are only part of a much larger picture when trying to understand the downstream circulatory effects impacted by periodontal disease. Investigation into differentiating microbial members that are cell-constrained vs. those that are transient may help uncover distinct patterns of dissemination and cell-associated microbiomes.

## Conclusion

Deepening the understanding of the relationships between periodontitis, metabolic endotoxemia, and blood microbiome will require additional study. The host-microbiome has been shown to impact neutrophil aging [103] and is required for steady-state hematopoiesis [103–105], implicating an intimately coordinated relationship exists between tissue and bone compartments. Both MyD88-dependent-TLR and Nod-Like-Receptors are involved in steady-state-hematopoiesis further underscoring the bacterial requirement, and quite likely LPS, for driving the regulation [105]. Another investigation into the microbial regulation of osteo-immunomodulatory effects suggest the oral microbiome, by selective use of an antibiotic mouthwash, specifically induces the pro-osteoclastic activity in the alveolar bone compartment [105, 106]. Together, these studies indicate that specific microbial-niches can modulate disparate immune-functions. The microbiomes that contain Gram negative bacteria and LPS content are capable of inducing and maintaining the populations of circulating neutrophil activity; careful evaluation is needed to improve our understanding of neutrophil activation, tolerance, and trained immunity in the context of systemic health. The circulating neutrophil, which we now understand can harbor bacteria acquired by patrolling local tissue sites that are colonized by pathogenic bacteria, can be primed or tolerized by exposure to different or subsequent LPS-phenotypes (defined by tissue-specific bacterial communities) and then traffic to the lymph or distal tissues with muted or exaggerated responses. It will be imperative to understand which tissue-specific-bacteria/communities impart the most meaningful systemic benefit or pathology to inform and improve treatment strategies for the constellation of inflammatory pathologies associated with periodontal disease.

## Author Contributions

All authors listed have made a substantial, direct, and intellectual contribution to the work and approved it for publication.

## Conflict of Interest

CZ was employed by Os Salutem LLC. The remaining author declares that the research was conducted in the absence of any commercial or financial relationships that could be construed as a potential conflict of interest.

## Publisher's Note

All claims expressed in this article are solely those of the authors and do not necessarily represent those of their affiliated organizations, or those of the publisher, the editors and the reviewers. Any product that may be evaluated in this article, or claim that may be made by its manufacturer, is not guaranteed or endorsed by the publisher.
